# Mycoplasmas under experimental antimicrobial selection: The unpredicted contribution of horizontal chromosomal transfer

**DOI:** 10.1371/journal.pgen.1007910

**Published:** 2019-01-22

**Authors:** Marion Faucher, Laurent-Xavier Nouvel, Emilie Dordet-Frisoni, Eveline Sagné, Eric Baranowski, Marie-Claude Hygonenq, Marc-Serge Marenda, Florence Tardy, Christine Citti

**Affiliations:** 1 IHAP, Université de Toulouse, INRA, ENVT, Toulouse, France; 2 UMR Mycoplasmoses of ruminants, ANSES, VetAgro Sup, University of Lyon, Lyon, France; 3 Asia-Pacific Centre for Animal Health, University of Melbourne, Melbourne, Victoria, Australia; University of Geneva Medical School, SWITZERLAND

## Abstract

Horizontal Gene Transfer was long thought to be marginal in *Mycoplasma* a large group of wall-less bacteria often portrayed as minimal cells because of their reduced genomes (ca. 0.5 to 2.0 Mb) and their limited metabolic pathways. This view was recently challenged by the discovery of conjugative exchanges of large chromosomal fragments that equally affected all parts of the chromosome via an unconventional mechanism, so that the whole mycoplasma genome is potentially mobile. By combining next generation sequencing to classical mating and evolutionary experiments, the current study further explored the contribution and impact of this phenomenon on mycoplasma evolution and adaptation using the fluoroquinolone enrofloxacin (Enro), for selective pressure and the ruminant pathogen *Mycoplasma agalactiae*, as a model organism. For this purpose, we generated isogenic lineages that displayed different combination of spontaneous mutations in Enro target genes (*gyr*A, *gyrB*, *parC* and *parE*) in association to gradual level of resistance to Enro. We then tested whether these mutations can be acquired by a susceptible population via conjugative chromosomal transfer knowing that, in our model organism, the 4 target genes are scattered in three distinct and distant loci. Our data show that under antibiotic selective pressure, the time scale of the mutational pathway leading to high-level of Enro resistance can be readily compressed into a single conjugative step, in which several Enro^R^ alleles were transferred from resistant to susceptible mycoplasma cells. In addition to acting as an accelerator for antimicrobial dissemination, mycoplasma chromosomal transfer reshuffled genomes beyond expectations and created a mosaic of resistant sub-populations with unpredicted and unrelated features. Our findings provide insights into the process that may drive evolution and adaptability of several pathogenic *Mycoplasma* spp. via an unconventional conjugative mechanism.

## Author summary

Genome downsizing is often viewed as a degenerative process of evolution. Such erosion has left current mycoplasmas with a minimal genome: for some species its size barely exceeds the amount of information needed for sustaining autonomous life. Despite such limitations, these simple bacteria showcase a baffling capacity for adaptation to complex environments such as that provided by the animal host. By using the enrofloxacin antibiotic as selective pressure, we performed a genome scale analysis of macro- and micro-events leading to antimicrobial resistance in mycoplasmas. Sexually competent cells were found to shortcut this process by using an unconventional mechanism of chromosomal transfer driving massive exchanges of DNA materials. Remarkably, this powerful mechanism was associated with a profound genomic reorganization that reshuffled parental features and created mosaicism. This finding emphasizes the extraordinary adaptability of some pathogenic *Mycoplasma* spp. and provides major insights into the processes that contribute to shaping the evolution of their minimal genome. While unconventional conjugative mechanisms are being documented in more complex bacteria, the reduced mycoplasma genome may provide a simplified model to study mosaicism and its role in bacterial evolution.

## Introduction

Over the past decade, advances in metagenomics have uncovered the fascinating richness and diversity of bacterial taxa. As free-living cells or as parasites, bacteria colonize an impressive array of ecosystems, from those offering ideal conditions to those too extreme to support most life forms. To better understand the forces that have shaped bacterial evolution, tremendous efforts have been invested in decrypting their genomes. One main outcome is that our traditional view of bacterial clonality and species boundaries is currently being challenged by the many facets of horizontal gene transfer (HGT), a key player of microbial diversification [1,2]. In this phenomenon, the role of mobile genetic elements (MGE) is central [3–6] and an increasing number of reports suggests that the transfer of these might only represent the tip of the iceberg [7–11]. Indeed, the conjugative transfer of large chromosomal fragments across genomes and their subsequent recombination might be more prominent and complex than first envisaged, with several new emerging mechanisms [7–11] that differ from the canonical Hfr- (or *oriT*-based) transfers. These latter ones were initially described in Hfr strains of *Escherichia coli* [12] and are initiated from an origin of transfer (*oriT*) integrated in the donor chromosome. *oriT*-based transfers are characterized by a gradient, with genes closer to the *oriT* being more reliably and more frequently transferred [13], mainly because of physical constraints applying on large molecules during transfer. Usually, in *oriT*-based transfers, a single region of the chromosome is transferred and incorporated.

HGT was long thought to be marginal in *Mycoplasma* (class *Mollicutes*), a large group of wall-less bacteria often portrayed as minimal cells because of their reduced genomes (ca. 0.5 to 2.0 Mb) and their limited metabolic pathways [14,15]. Despite this simplicity, several mycoplasma species are important pathogens of human and a wide range of animals [15,16]. This situation reflects our failure in providing efficient preventive and therapeutic strategies and is due to the mycoplasma astonishing capacity to face the challenging host-environment, escape the immune response and develop antimicrobial resistance (AMR) [14,17,18]. Mycoplasmas live in close contact with their immunocompetent hosts on which they rely for nutrients and, in these wall-less bacteria, several loci have been selected over the course of their evolution that generate surface diversity [17,19]. These encode for a broad range of molecules that are key in host-interactions [17,20] and in escaping the host humoral response. The variation in expression and structure of these products relies on sophisticated genetic systems that combined large gene repertoires with high frequency, stochastic mutations or specific-recombination. In mycoplasmas, these systems account for extensive intra-clonal and inter-strains variability [17]. More recently, comparative genomic studies have uncovered the occurrence of massive HGT in between phylogenetically distant mycoplasma species, a phenomenon that may counteract erosion of the reduced mycoplasma genome and account for genome plasticity [21,22]. This finding was further supported by experimental data showing the conjugative exchange of large chromosomal fragments in *Mycoplasma agalactiae*, an important ruminant pathogen and a model organism [9]. Congruent *in silico* and *in vitro* data further demonstrated that these transfers equally affected all part of the chromosome via an unconventional mechanism, so that the whole mycoplasma genome is potentially mobile. While this has been formally demonstrated for *M*. *agalactiae* and *M*. *bovis*, increasing evidences point towards HGT also occurring in other species such as in *M*. *pulmonis* [23], *M*. *genitalium* [24] and in other genera of the class *Mollicutes* such as in *Ureaplasma* or *Spiroplasma* [25–28]. HGT may have tremendous impact on the long and short-term evolution and adaptability of these minimal bacteria but has yet to be explored. Using antibiotics as selective pressure would offer a powerful approach for testing this question *in vitro*; at the same time understanding the emergence of antibiotic resistance in pathogenic mycoplasmas is of primary importance for public health [18,29].

The horizontal dissemination of MGE carrying AMR genes, within and across bacterial species is one main determinant of the antibiotic crisis [30,31]. In mycoplasmas, the role of HGT in acquiring AMR has long been ignored mainly because of (i) the paucity in MGEs and the total lack of known conjugative plasmids that could disseminate AMR genes and (ii) the scarcity of appropriate genetic tools which, combined to the mycoplasma fastidious culture hampered testing the hypothesis under laboratory conditions. The main genetic pathway described so far for the emergence of AMR in these organisms is the occurrence, selection and fixation of chromosomal mutations in target genes [18,29]. For instance, mutations conferring quinolone resistance have been reported in pathogenic mycoplasma species [32–34] as well as in several other bacterial taxa [35,36]. Quinolones, an important class of antibiotics effective on the wall-less mycoplasma cell, exert their antibacterial effect by preventing the DNA gyrase and the topoisomerase IV from unwinding and duplicating DNA [37,38]. Mutations occurring in genes encoding DNA gyrase subunits, *gyr*A and *gyrB*, and/or topoisomerase IV subunits, *parC* and *parE*, result in structural changes in the respective enzyme that limit antibiotic fixation, with the QRDR (Quinolone Resistance Determining Region) of GyrA and ParC being most often affected at key positions (amino acids 83 for GyrA and 80, 84 for ParC according to *E*. *coli* numbering) [36].

In this study, we experimentally explored the impact of conjugative chromosomal transfer on mycoplasma evolution and adaptation using a fluoroquinolone, the enrofloxacin (Enro), as selective pressure and *M*. *agalactiae*, as a model organism. For this purpose, we first generated spontaneous isogenic mutants displaying different combination of mutations in *gyr*A, *gyrB*, *parC* and *parE* together with various level of resistance to Enro. We then tested whether these mutations can be acquired by a susceptible population via conjugative chromosomal transfer knowing that in several *Mycoplasma* spp., including our model organism, the 4 target genes are located in distinct chromosomal loci; in *M*. *agalactiae* these are separated by at least 250 kb, with *parE* and *parC* being part of a same operon. Under antibiotic selective pressure, spontaneous mutants emerged stepwise following a similar pathway with HGT acting as an evolutionary accelerator that reshuffled genomes and created a mosaic of resistant sub-populations with unpredicted and unrelated features. Our findings provide insights into the process that may drive evolution and adaptability of several pathogenic mycoplasma species and bring into the light an unconventional conjugative mechanism.

## Results

### Selection of spontaneous mycoplasma mutants, highly resistant to enrofloxacin

**Fig 1:**
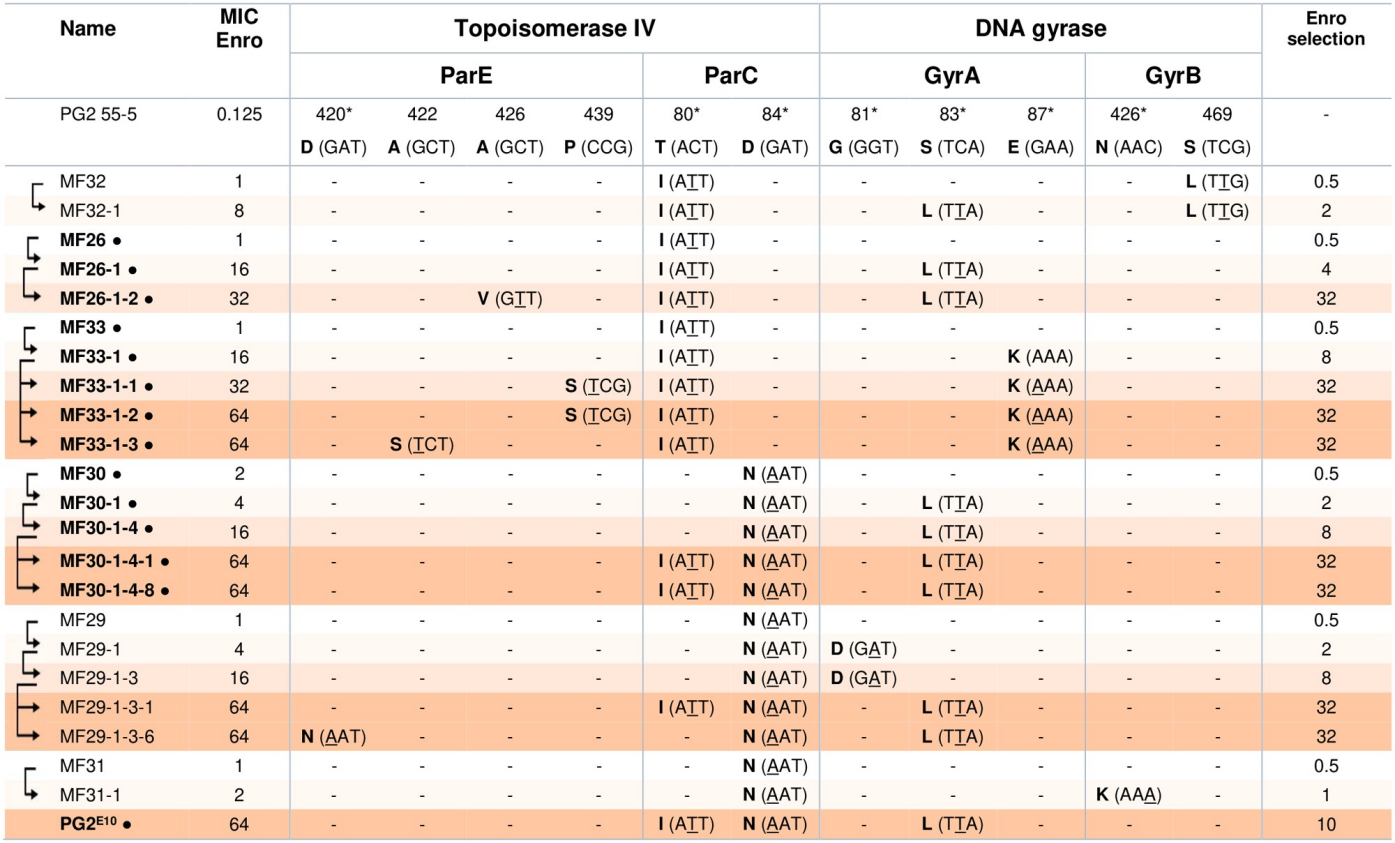
Accumulation of mutations in the QRDR of *parE*, *parC*, *gyrA* and *gyrB* correlates with the increase in the level of resistance to enrofloxacin in *M*. *agalactiae*. Isogenic lineages composed of mutants with increasing level of resistance to Enro were generated using the *M*. *agalactiae* parental clone, PG2 55–5 (NC_009497.1). Within each isogenic lineage, the mutant’s relationships are indicated with black arrows on the left side. Amino-acid changes in QRDR regions of *par*E, *par*C, *gyr*A, and *gyr*B are indicated according to *E*. *coli* numbering along with their corresponding codons in which the mutated nucleotides are underlined. *: amino acid belonging to the QRDR; -: Identical to the parental PG2 55–5. ●: mutants further subjected to whole-genome sequencing. The enrofloxacin concentration is given in μg.mL^-1^.

Prior to testing the impact of Mycoplasma Chromosomal Transfer (MCT) on AMR acquisition (see below), we analysed the evolutionary pathway leading to high resistance to enrofloxacin (Enro) in a set of spontaneous mutants derived from PG2 55–5. For this purpose, six lineages namely MF26 and MF29 to MF33 (Fig 1), were generated by rounds of single-colony bottleneck selection on solid medium containing stepwise concentrations of Enro (0.5 to 32 μg·ml^-1^, with a two-fold step interval). At each step, single colonies were picked and analysed as described in Materials and Methods. Overall, 22 individual isogenic clones with MIC (Minimal Inhibitory Concentration) values ranging from 1 to 64 μg·ml^-1^ were selected and their *parE-*, *parC-*, *gyrA-* and *gyrB-*QRDR (Quinolone Resistance Determining Region) [39,40] were sequenced, directly from the chromosome.

Sequence data revealed the occurrence of 1 to 3 single-point mutations in each of the 22 Enro^R^ clones (Fig 1), with a total of 11 SNPs (Single Nucleotide Polymorphisms) detected in distinct positions, none being silent. All had at least one mutation within *parC* which always corresponded to a transition, C>T or G>A, and resulted in amino-acid changes at codon 80 and/or 84 of the QRDR (further designated *parC*_80_ and *parC*_84_, respectively). In 68% of the mutants, an additional point mutation was present in *gyrA* that also resulted in codon change within the QRDR, most often at codon 83 and in some cases, at codon 87 or 81 (further designated *gyrA*_83_, *gyrA*_87_ and *gyrA*_81,_ respectively). Finally, additional mutations were occasionally found in *parE* and *gyrB*, affecting the canonical QRDR only in mutants MF29-1-3-6 and MF31-1, in *parE* codon 420 (*parE*_420_) and *gyrB* codon 426 (*gyrB*_426_), respectively (Fig 1).

Within each lineage, the MIC value was shown to increase over the stepwise selection process together with the number of accumulated mutations. This is illustrated in Fig 1, with for instance the MF26 lineage acquiring a new mutation at each round of selection, in *parC*, *gyrA* and then *parE*, concomitantly to a MIC increase from 1 to 32 μg·mL^-1^. This pattern was observed for all lineages, with a few cases of one-step MIC increase that were not linked to an additional mutation in the sequenced regions (Fig 1, MF30-1>MF30-1-4 and MF29-1>MF29-1-3).

Overall, Enro^R^ mutations accumulate following a common pathway, emerging first in *parC*, then in *gyrA* (or for MF31 in *gyrB*) and last in *parE*. From these data, the contribution of *parE* mutations did not seem as critical towards resistance as those occurring in *parC* and *gyrA*, some mutants having a high MIC value and no *parE* mutation (see for instance MF30-1-4-1).

Passaging of PG2 55–5 in broth medium containing increasing concentration of Enro, without intermediate rounds of sub-cloning onto solid medium, resulted in a selected PG2^E10^ population having a MIC of 64 μg·ml^-1^ and 3 SNPs located in *parC*_80_, *parC*_84_ and *gyrA*_83_, as in MF30-1-4-1, MF30-1-4-8 and MF29-1-3-1 (Fig 1).

### Mutations conferring increasing resistance to enrofloxacin preferentially accumulate in type II topoisomerase genes

**Fig 2:**
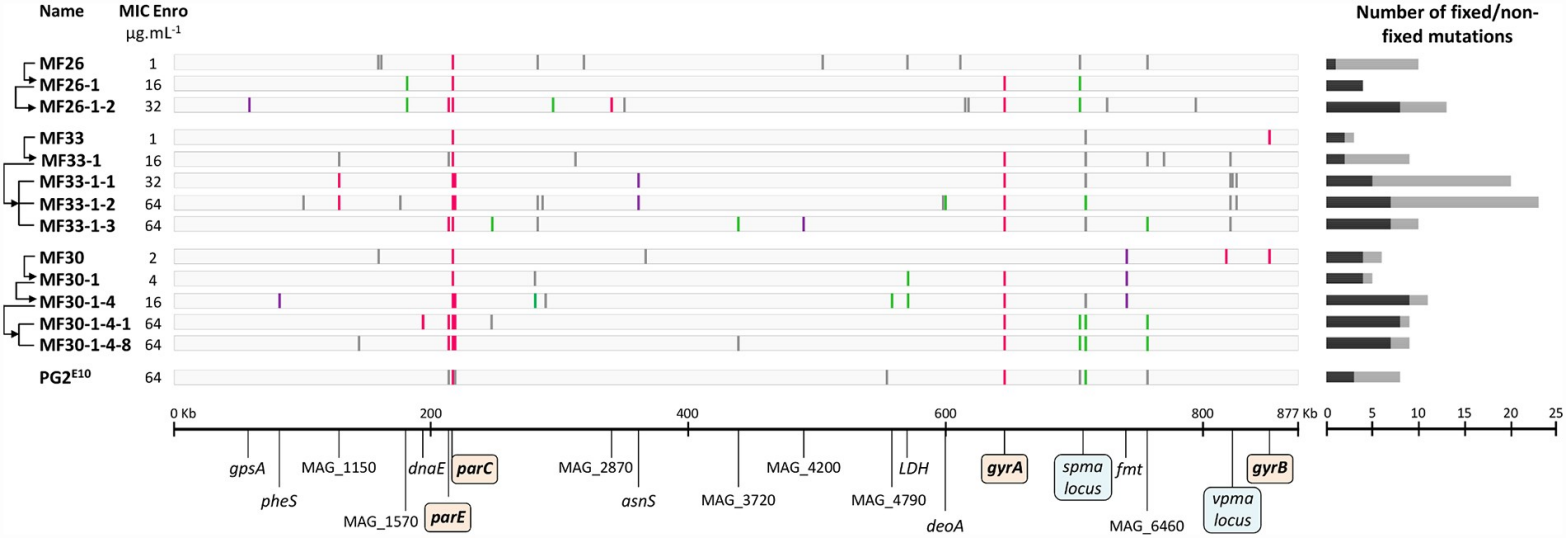
Whole genome analysis of spontaneous *M*. *agalactiae* Enro^R^ mutants. Nature and localisation of mutations detected in genomes of Enro^R^ mutants. Genomes are represented by horizontal grey rectangles starting on the left by the origin of replication. Mutations (SNPs and indels) are indicated by vertical lines in comparison to the reference PG2 55–5 genome (NC_009497.1). Black arrows on the left side indicate the parental relationships within each isogenic lineage, with the enrofloxacin MIC indicated in μg.mL^-1^. Fixed mutations (≥ 95% of reads) are represented by coloured lines (synonymous SNP in violet, non-synonymous SNP in pink and indel in green) and grey lines represent non-fixed mutations (< 95% of reads). Genes impacted by fixed mutations are indicated below, by their Genbank locus tag, or gene name when defined. Enrofloxacin target genes and variable loci are underlined in orange and blue, respectively. The total number of mutations for each mutant (fixed mutations in black, non-fixed in grey) is depicted by a diagram on the right side.

Some discrepancies between the number of mutations and the MIC values (see above) raised the question of whether the level of resistance may be modulated by mutations occurring outside the QRDR regions. To address this issue, the genome of 13 clones belonging to 3 independent and representative lineages (MF26, MF33, and MF30) was fully sequenced by Illumina with a mean coverage of 3100X. SNPs and indels were identified by variant calling analyses using the PG2 55–5 parent clone as reference (Fig 2). For 6 mutants (MF33, MF30, MF33-1, MF30-1-4, MF30-1-4-1, and MF30-1-4-8), WGS (Whole Genome Sequencing) data revealed the occurrence of the SNPs *parE*_*86*_, *parE*_*112*_, *parC*_*291*_, *parC*_*547*_, *gyrB*_*29*_ *and gyrB*_*278*_ (S2 Table) in the 3 target genes; these SNPs are located outside the region previously sequenced with the Sanger method and thus are outside the QRDR. Data also suggested that *gyrB* mutations found in MF30 and MF33 lineages may have a negative impact on the further selection of highly resistant mutants. Indeed, the *gyrB*_29_ and *gyrB*_218_ mutations were only detected in the founders, MF33 and MF30. Reversion of these mutations in progenies coincided with the emergence of mutations in *gyr*A which ones were further transmitted under increasing antibiotic pressure, a series of events that may reflect an epistatic phenomenon. As well, the reversion of *parC*_*291*_ and *parC*_*547*_ mutations in MF30 lineage was accompanied by the appearance in more resistant progenies of new mutations in *parC* and *parE*. Overall, these abrupt changes may be due to the constraints imposed by the interdependence of *gyrA* and *gyrB* or *parE* and *parC* subunits in forming a functional DNA gyrase or topoisomerase IV, respectively, that will best withstand the antibiotic pressure. A few mutations (SNPs and indels) were also detected outside of the classical quinolone target genes (Fig 2 and S2 Table), with most occurring in homopolymeric tracts that are known as being prone to high frequency insertion-deletion [17]. For instance, nt-707306 and/or nt-711627 both underwent a C deletion within a polyC of the so-called *spma* locus which encode phase variable membrane proteins [41]. As well, the MF33-1-1 and -1-2 siblings both contained sub-populations displaying a large number of SNPs (14 and 11) within the highly variable *vpma* locus [42]. Overall, sequenced genomes contained from 3 to 23 mutations, with a mean of 10.2 ± 5.8 mutations, with approximately half being fixed in the population (present in ≥95% of the reads).

In parallel, the genome of the Enro^R^ PG2^E10^ was analysed and a total of 8 mutations were detected, 3 fixed and 5 non-fixed (present in <95% of the reads and further refer as polymorphic sites), when compared to the parental strain (Fig 2 and S2 Table). As expected, 3 SNPs were found in the Enro target genes, *parC*_80_, *parC*_84_, *gyrA*_83_, and an additional one was detected in *parE*_*112*_, outside of the QRDR initially sequenced by Sanger (see above). Among the 3 studied lineages, this combination of 4 SNPs was only found in MF30-1-4-1 and MF30-1-4-8, which MIC of 64 μg·ml^-1^ is identical to that of PG2^E10^. The other 4 mutations occurred outside the target genes and correspond to either highly variable loci (see above) or to mutations occurring only in minor subpopulations (polymorphic sites). Based on competition fitness assays, these mutations did not appear to impose a cost on the PG2^E10^ fitness (w = 1.03 ± 0.10) when compared to PG2 55–5 parent (see Materials and methods).

### Horizontal chromosomal transfer of multiple, distant Enro^R^-mutations accelerates AMR dissemination

**Fig 3:**
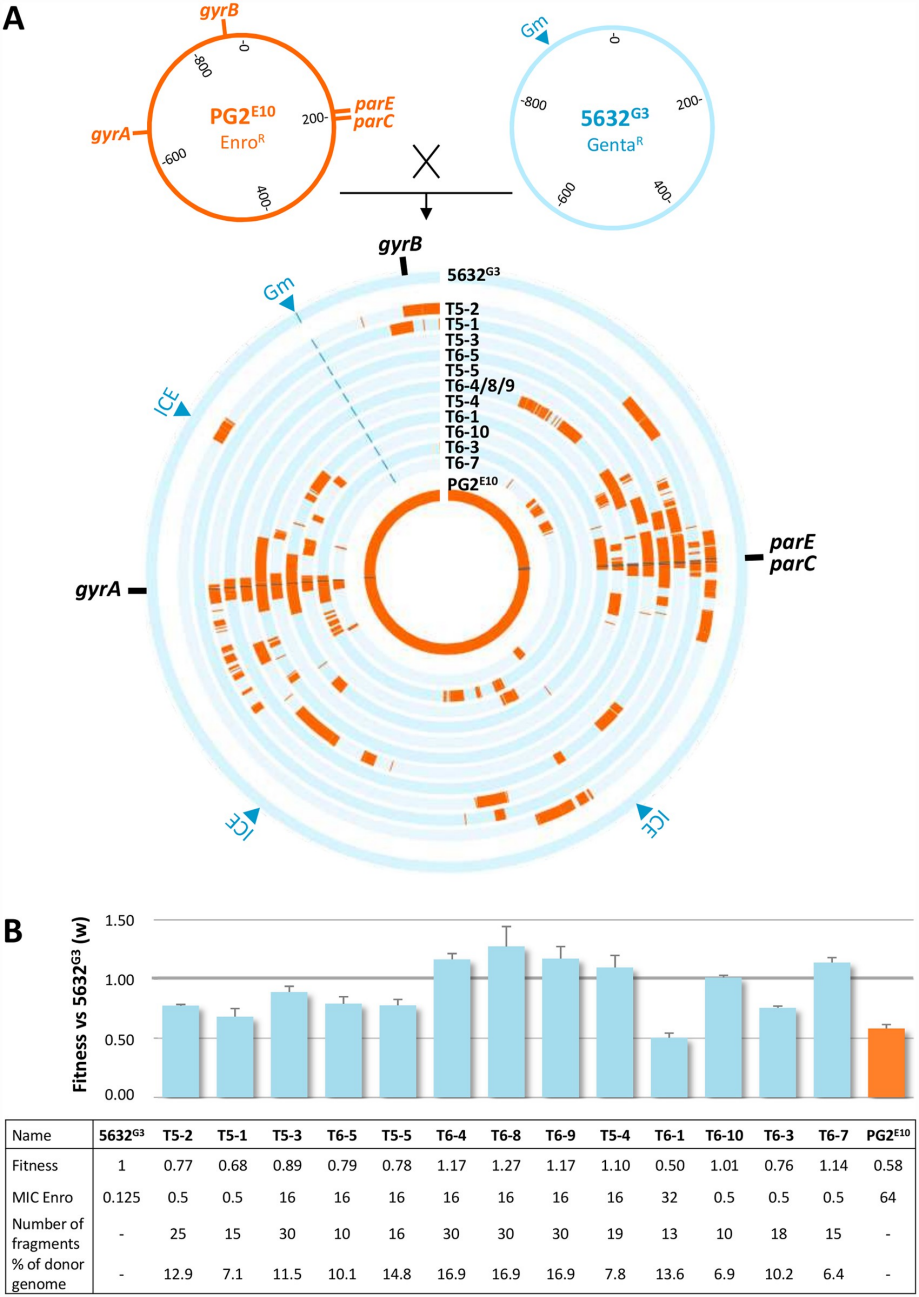
Mycoplasma transconjugant genomes and relative fitness. (A) Mating experiments were conducted using the Enro^R^ PG2^E10^ as donor and, the Enro^S^, gentamicin-tagged 5632^G3^ as recipient. Individual transconjugants were selected on solid media containing enrofloxacin and gentamicin and their genomes further sequenced by Illumina (see Materials and methods). Parental and transconjugant genomes are represented as concentric circles with PG2^E10^- and 5632^G3^-specific sequences indicated in orange and blue, respectively. The location of the gentamicin resistance marker (Gm) and of the Enro target genes *parE*, *parC*, *gyrA* and *gyrB* are shown on the periphery, with vertical black lines indicating the position of mutations relative to PG2 55–5. ICE relative positions in the 5632 genome are indicated by blue arrows. Strains and transconjugants are from inner to outer circle: PG2^E10^, T6-7, T6-3, T6-10, T6-1, T5-4, T6-4/8/9, T5-5, T6-5, T5-3, T5-1, T5-2 and 5632^G3^. (B) Transconjugants relative fitness (w) compared to the 5632^G3^ recipient strain is represented by histogram bars. The table summarizes the w values, the enrofloxacin MIC (in μg.mL^-1^), the number of PG2^E10^ fragments and the percentage of donor genome for each transconjugant. The percentage of donor genome was estimated by the total size of the PG2^E10^ transferred fragments divided by the size of the PG2^E10^ genome.

Our hypothesis is that horizontal conjugative chromosomal transfer may act as a driving force of mycoplasma short-term evolution. With this in mind, we tested whether multiple, distant chromosomal Enro^R^ point-mutations can be simultaneously transferred by HGT from a resistant to a susceptible strain and further selected in presence of the antimicrobial. For this purpose, two independent mating experiments (T5 and T6) were performed using as donor the Enro^R^ PG2^E10^ mutant (see above) which displayed the highest MIC (64 μg.ml^-1^) but the smallest number of fixed mutations (see Fig 2 and S2 Table). As recipient, we choose the 5632^G3^ clone previously derived from the Enro^S^ 5632 strain (MIC = 0.125 μg.mL^-1^) and in which the gentamicin-resistance marker (Gm) is stably inserted as a proxy [9] (see Materials and methods) (Fig 3A). Transconjugants were selected on solid media containing 50 μg·mL^-1^ of gentamicin in addition to Enro at concentrations ranging from 0.25 to 8 μg·mL^-1^ (equal to 2 to 64 fold the MIC 5632^G3^). Repeated attempts consistently yielded transconjugants colonies on solid medium containing 0.25 μg·mL^-1^ of Enro (except for T5-5 obtained at 0.5 μg·mL^-1^) with a low frequency ranging from 2.7.10^−11^ to 7.2.10^−8^ transconjugants per donor-CFU, depending on the 5632^G3^:PG2^E10^ initial ratio (1:10 or 10:1, respectively, see Materials and methods).

A total of 18 individual transconjugants were then picked and subjected to a series of PCR assays. These targeted the Gm marker and 11 distant loci that are distributed around the genome and discriminate 5632 from PG2 (S1 Fig, S1 Table). Of these, 8 were previously described [9] and 3 were specifically designed in this study to distinguish PG2-*parC*, -*gyrA* and -*gyrB* from their 5632 counterparts. PCR data indicated that the transconjugant genotypes were a composite of PG2 and 5632 genomes (S1 Fig) except for T6-7 which was further shown by sequencing to have a chimeric *gyrA* (see below). They further designated 5632^G3^ as the recipient chromosome, with a majority of the PCR products being 5632-specific and the Gm marker constantly detected at the same position, as in the parent. This finding was in agreement with our previous data showing that chromosomal transfers always occurred from PG2 (donor) to 5632 (recipient) [9]. Overall, 10 distinct PCR profiles were observed, with several transconjugants sharing identical profiles (S1 Fig).

Whole Genome Sequencing (WGS) by Illumina was performed with a subset of 13 transconjugants that were selected (i) to represent each of the 10 PCR profiles identified above and (ii) to include transconjugants with identical PCR profiles that were generated during independent (T5-4 and T6-1) or during the same (T6-4, -8, -9) mating experiments. Sequence data confirmed that all displayed the 5632^G3^ chromosome as genetic background designating the corresponding strain as the recipient (Fig 3A). Further analyses demonstrated the systematic transfer of PG2^E10^ donor remote Enro target-genes containing (i) the mutated *parE/parC* operon (10/13 transconjugants) together with either the mutated-*gyrA* or the wild-type *gyrB* (wt) or (ii) the mutated *gyr*A alone (3/13) (Fig 3A). A close-up image of these regions is depicted in Fig 3A and shows that two fixed mutations, corresponding to *parC*_80_ and/or *gyrA*_83_, were always associated to the transfer. Of note, the chimeric structure of T6-7 *gyrA* explains the PCR result obtained above (S1 Fig).

**Fig 4:**
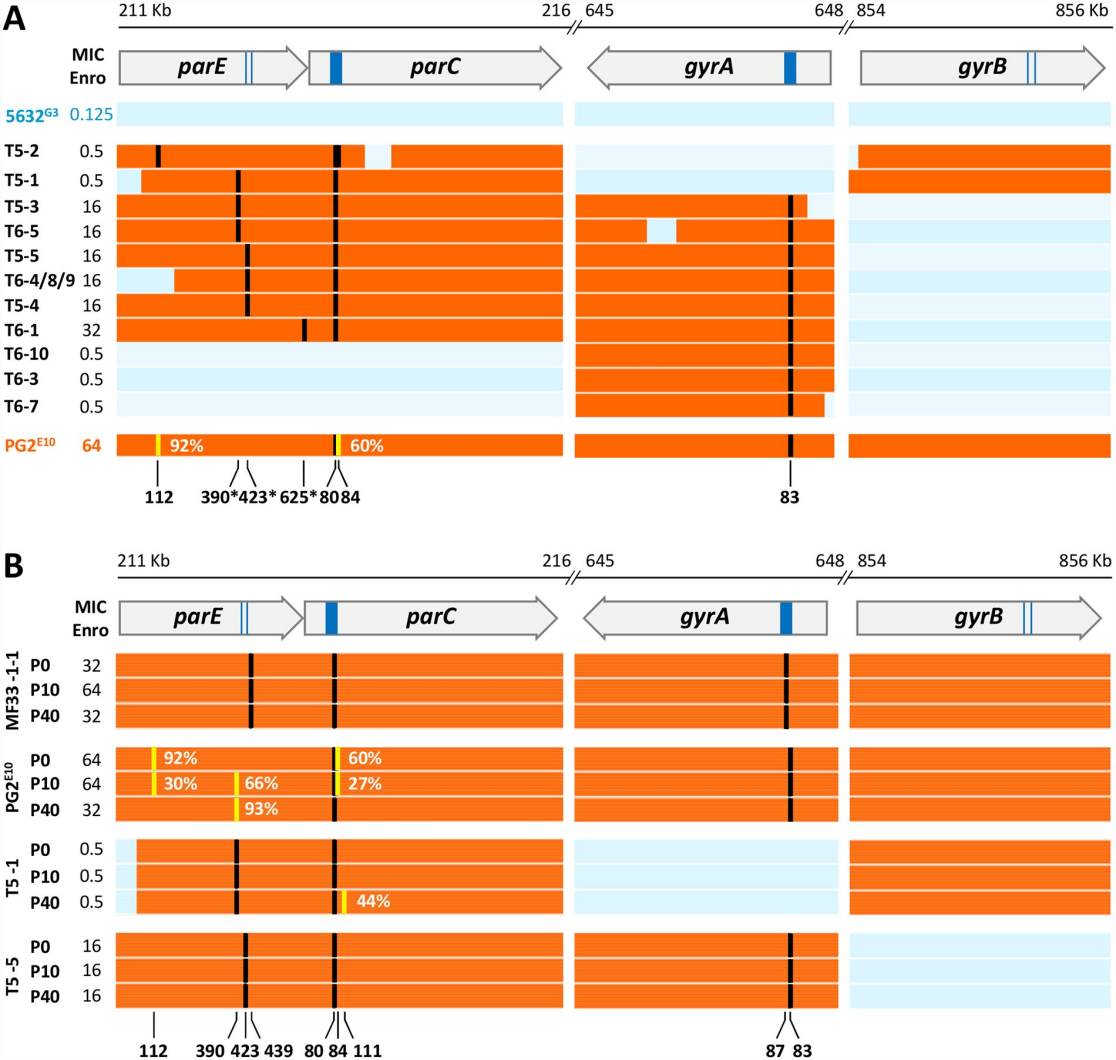
Comparison of *parE*, *parC*, *gyrA* and *gyrB* gene sequences in transconjugants and in Enro^R^ mutants. (A) Schematic representing the 4 Enro target genes (*parE*, *parC*, *gyrA* and *gyrB*) in the 13 transconjugants analysed in Fig 2. Their coding sequences are represented by an open arrow with the dark blue box correspond to the QRDR. The enrofloxacin MIC values are indicated in μg.mL^-1^. PG2- and 5632-specific sequences are represented in orange and blue, respectively. Mutations are indicated in black (fixed mutations) or yellow (non-fixed mutations) and their corresponding amino-acid positions are indicated based on *E*. *coli* numbering. Mutations not detected in the PG2^E10^ donor strain are indicated by a star. (B) Schematic representing the 4 Enro target genes *parE*, *parC*, *gyrA* and *gyrB* (as above) of two spontaneous mutants (MF33-1-1 and PG2^E10^) and two transconjugants, (T5-1 and T5-5) after serial propagation in broth culture without selective pressure. P0, P10 and P40 correspond to passages 0, 10 and 40 in the absence of enrofloxacin selection pressure, with P10 and P40 corresponding to approximately 165 and 605 generations respectively.

Of the 13 transconjugants analysed, 10 had received *parE* sequences from the donor. One, T5-2, had acquired two mutations that were pre-existing in PG2^E10^ sub-populations (92% and 60% of the reads, respectively), *parE*_*112*_ and *parC*_*84*_. The remaining 9 transconjugants displayed one mutation in *parE*, not previously detected in the donor, that was either (i) an insertion of 3 nt resulting in adding an Ala residue at codon 390 or (ii) a non-synonymous SNP corresponding to codon 423 or 625. At least, mutations corresponding to codons 390 and 423 were independently confirmed by direct genome sequencing. Whether the occurrence of these mutations in some transconjugants reflects the heterogeneity of the PG2^E10^ donor population, with sub-populations being selected here, or whether they have arisen independently after transfer is not known. Of note, T5-2 *parC* and T6-5 *gyrA* were more chimeric than in other transconjugants as if multiple recombination events have occurred to produce mosaic genes composed of 5632 and PG2 intermingled sequences (Fig 4A).

Interestingly, none of the transconjugants accumulated all 4 SNPs described for the PG2^E10^ in the Enro target genes. As well, none reached the 64 μg·mL^-1^ MIC of the PG2^E10^ parental strain but their individual MIC value that ranged from 0.5 to 32 μg·mL^-1^ (Fig 4A) was always higher than the concentration used for selection (0.25 μg·mL^-1^). More specifically, transconjugants having concomitantly acquired the two distant PG2^E10^ loci containing the mutated *parE-parC* and the mutated *gyrA* had the highest MIC (16 μg·mL^-1^ to 32 μg·mL^-1^). In T5-1 and T5-2 that displayed the lowest MIC value (0.5 μg·mL^-1^), the mutated *parE-parC* were co-transferred with the wild-type PG2^E10^*-gyrB* instead of the mutated PG2^E10^*-gyrA*. Since donor and recipient *gyrB* allelic sequences differ slightly, this event introduced amino acid changes in GyrB when compared to the parental 5632-background (S5 Table). PCR genotyping indicates that this same combination was also observed in T6-2, a transconjugant derived from the same partner but in an independent mating experiment (see S1 Fig). Finally, it is interesting to note that the co-transfer of the PG2^E10^ mutated-*gyrA* and its wt-*gyrB* was never observed. In agreement with data obtained with the spontaneous mutants, the transfer of mutated-donor *gyrA* only was not sufficient to confer the recipient strain with the Enro^R^ phenotype.

Overall, mutations conferring resistance to Enro with MIC values ranging from 16–32 μg.mL^-1^ could be acquired by a susceptible population within one mating experiment via HGT, while reaching the same levels of Enro^R^ MIC through spontaneous mutations would have required approximately 100 generations and multiple passages under selective pressure.

### Antimicrobial selection drives the emergence of unrelated genotypes via unconventional HGT

The co-transfer of multiple loci and the possible occurrence of additional macro- and micro-heterogeneities were addressed in the 13 sequenced transconjugants. Reconstruction of the composite-genomes was performed as previously described with some minor modifications [9] (see Materials and methods). Briefly, reads generated by NGS (Next Generation Sequencing) were mapped onto the 5632 and PG2 reference genomes and reads perfectly matching to one or the other genome were retained. Analyses of the reconstructed genomes and more specifically of the PG2 inherited sequences confirmed that other fragments, unrelated to topoisomerase genes carrying Enro^R^ mutations, were also exchanged (Fig 3A and S2 Fig). This resulted in complex mosaic genomes, containing an average of 18 ± 7 PG2^E10^ fragments (Fig 3B) which size varied from 77 bp to 53429 bp. All transconjugants display distinct patterns of transferred fragments, except for 3, which had strictly identical genome sequences (S2 Fig). These clones, namely T6-4, -8 and -9, were all selected from the same mating experiment and are most likely the result of the expansion of a single transconjugant as all were shown to be fitter than the parent or than other transconjugants produced during the same mating (i. e. T6-5 and T6-1) (Fig 3B). Competitive culture assays also indicated that there was no correlation between the number of fragments or the overall DNA amount that was exchanged and the fitness level (Fig 3B), with some combinations imposing a fitness cost while other conferring a fitness benefit.

Overall, the most frequently transferred regions were clustered within 20 kb around the selective Enro^R^ determinants, but distant loci were also exchanged in all transconjugants. This suggested that multiple events of genomic replacements by recombination have occurred simultaneously. Although the PG2 and 5632 genomes are highly syntenic, some genes or regions are only present in one strain. Thus, in some cases, replacement of 5632 recipient genome by a PG2 fragment resulted in the loss or in the gain of strain-specific genes. On average, 13 ± 11 5632-specific genes were lost for 6 ± 4 PG2-specific genes that were gained (S3 Table). One extreme case of replacement resulted in the deletion of a large region (ca. 22 genes, 27 Kb) which contained an integrated conjugative element (ICE) specific to 5632 and not present in PG2 [43]. This was observed in T5-2, T5-5, T6-1 and T6-7 transconjugants (Fig 3A, S3 Table) where the loss of the 22 genes was not due to the ICE excision but to recombination events occurring at homologous sites on each side of the ICE.

In addition, micro-complexity events were observed, with transconjugants displaying short PG2-inherited fragments (180 ± 29 nt) that were defined by only one or two PG2-specific variations (SNPs or indels), and/or the occurrence within PG2-inherited fragments of short 5632 fragments defined by one or two 5632-specific variations.

Overall, chromosomal exchanges by recombination of large or small fragments were shown to often occur within a coding sequence, resulting in chimeric PG2/5632 genes as illustrated above for *parC* and *gyrA*. On average 20 ± 7 genes were mosaic to various degrees, for each transconjugant.

### *In vitro* multiplication without selection pressure reveals fine-mutational tuning

To evaluate the stability of the Enro^R^ spontaneous mutants and transconjugants in absence of selective pressure, two different spontaneous mutants, namely, MF33-1-1 (MIC = 32 μg·mL^-1^) and PG2^E10^ (MIC = 64 μg·mL^-1^), and two different transconjugants T5-1 (MIC = 0.5 μg·ml-1) and T5-5 (MIC = 16 μg·mL^-1^), were submitted to serial passages in broth medium 40 times (P0 to P40). WGS was performed with DNA extracted at P10 and P40 that correspond to approximately 165 and 605 generations, respectively (see Materials and methods). Based on comparative analysis, the genomes of the 2 mutants and the 2 transconjugants were remarkably stable over this period, in agreement with the overall stability of their MIC over passages (Fig 4B and S4 Table).

Interestingly, the two non-fixed mutations pre-existing in PG2^E10^ *parE* and *parC*, respectively, gradually faded in favour of the wildtype (Fig 4B): one was detected in *parE*_112_ in 92%, 30% and 0% of the reads and the other in *parC*_84_ in 60%, 27% and 0% of the reads, at P0, P10 and P40 respectively. Concomitantly, an indel emerged in *parE*_390_ at P10 (66%) and P40 (93%) that resulted in the insertion of an Ala residue. Interestingly, this same insertion occurred in 3 transconjugants: T5-1, T5-3 and T6-5, in which it was fixed. As shown in Fig 4A, the absence of mutation in *parC*_84_ in all but one transconjugant coincides with the presence of mutations in *parE*. Altogether, these data suggested that mutations in *parC* might have a slight fitness cost that tended to be compensated over passages by the introduction of mutations in *parE*, the functional partner of *parC*. Of note, other polymorphic sites were observed elsewhere in the genome during passages. In particular, MF33-1-1 with 9 polymorphic sites at P10, displayed the highest number of non-fixed mutations (excluding those in *vpma* locus) most of which (6/9) being lost at P40 (S4 Table).

**Fig 5:**
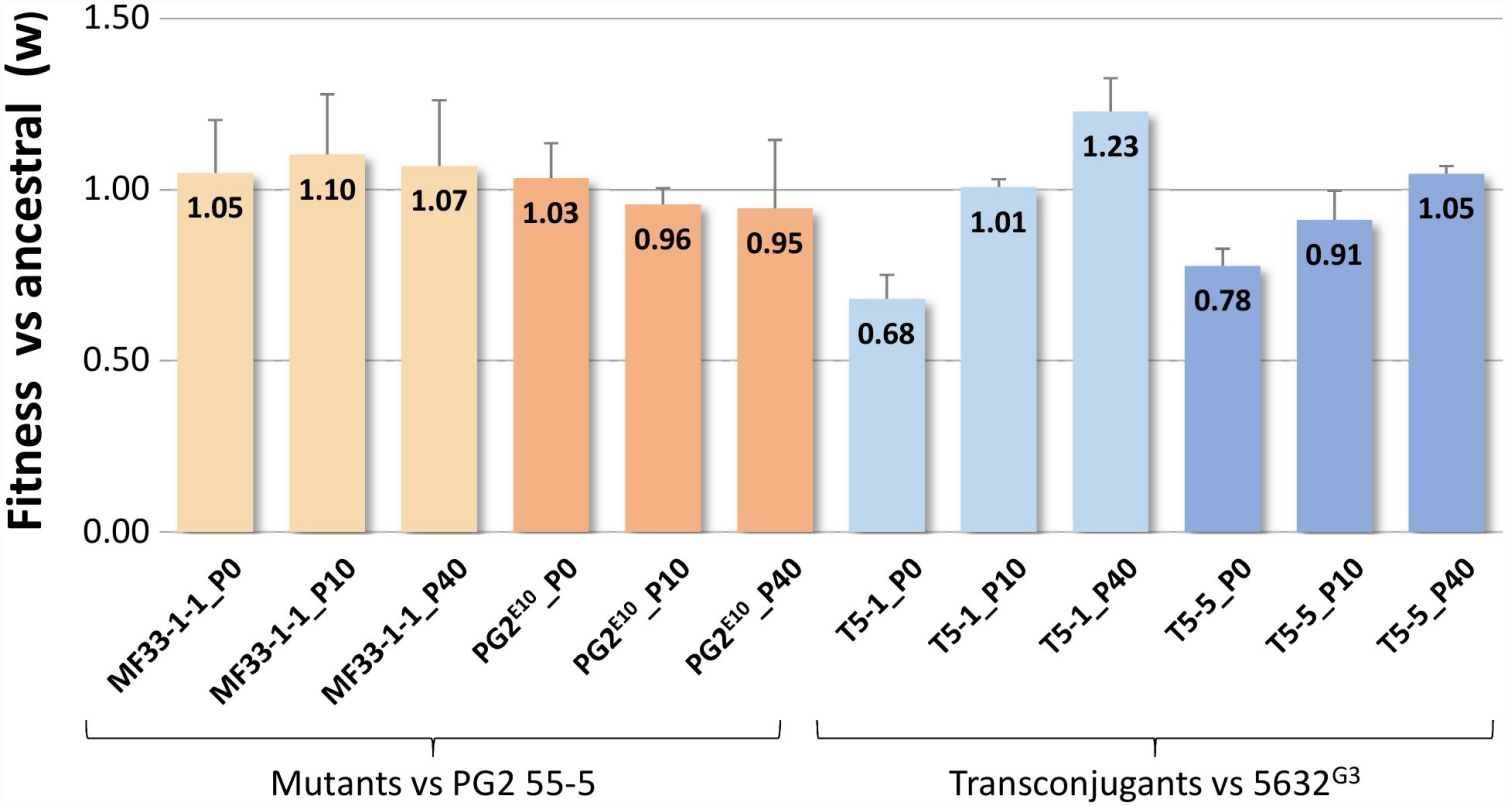
Relative fitness of mutants MF33-1-1 and PG2^E10^ and of transconjugants T5-1 and T5-5 over passages. The relative fitness (w) of mutants and transconjugants versus their ancestor strain, respectively, PG2 55–5 and 5632^G3^ was defined by pairwise competition assays (see Materials and methods). P0, P10 and P40 correspond to passages 0, 10 and 40 in the absence of enrofloxacin selection pressure, with P10 and P40 corresponding to approximately 165 and 605 generations respectively. At least three replicates were performed for each assay; the mean value and the standard deviation (SD) are indicated on the graph.

We then investigated the fitness of the two mutants and two transconjugants over passages in broth medium. Data presented in Fig 5 indicated that mutants MF33-1-1 and PG2^E10^ displayed a fitness similar to that of the PG2 55–5 ancestor (P0) that remained stable over passages (P10 and P40). In contrast, the fitness of transconjugants T5-1 and T5-5 at P0 was reduced by 30 to 20%, respectively and increased over successive passaging to reach 120 and 100% when compared to the recipient strain, 5632^G3^. WGS showed that a few different, polymorphic sites accumulate over passages in both the mutants and the transconjugants, without any obvious link to fitness (see S4 Table).

## Discussion

Over the past decade, HGT has increasingly attracted attention and is now recognized as a main driver of microbial innovation, with conjugation as one prominent mechanism [2,44,45]. Yet, knowledge regarding the mechanisms and impacts of HGT in mycoplasmas is very limited, with only a few publications dedicated to this topic [9,21–23]. By combining next generation sequencing to classical mating and evolutionary experiments, this study uncovered the role of an unconventional mechanism of HGT in generating mosaic genomes in *M*. *agalactiae*. Under evolutionary experimental conditions, this phenomenon acted as an accelerator of AMR dissemination by providing susceptible mycoplasma cells with the ability to rapidly acquire, from pre-existing resistant populations, multiple chromosomal loci carrying AMR mutations.

In *M*. *agalactiae*, high-level of Enro resistance can be reached via the emergence of spontaneous chromosomal mutations during propagation with increasing concentrations of the antimicrobial, as shown for other *Mycoplasma* species [32,34,46]. The comparison of several, independent lineages indicates that these mutations accumulate following a similar trajectory: first in *parC* resulting in a 8 to 16-fold increase in resistance, followed by additional mutations in *gyrA* to reach up to 128-fold increase. Higher resistance levels (up to 500-fold) were further achieved by combining either two mutations in *parC* with one in *gyrA* or, one mutation in each with one or more mutations in *parE*. WGS data further showed that only very few other mutations were selected and fixed outside of these genes, none that could account for the resistance phenotypes. Whether these played a role in counterbalancing a potential fitness cost during the selection process is not known but mutations in type II topoisomerase genes had no effect on PG2^E10^ fitness *in vitro* when compared to the ancestor strain (w = 1.03 ± 0.10). Overall, accumulation of fixed mutations in type II topoisomerase genes and high levels of resistance were reached over several weeks of propagation, after approximately 200 or 100 generations depending on whether selection was performed with or without bottleneck selection, respectively. A limited number of reports addressed evolutionary trajectories of fluoroquinolones resistance in bacteria and each identified species-specific mutational trajectories with identical target-site mutations emerging in different order [47–49]. In *M*. *agalactiae*, the convergent outcome of parallel independent experiments strongly suggested intermolecular epistatic interactions between DNA topoisomerases in the mechanism of fluoroquinolone resistance.

The time scale of the mutational pathway leading to high-level of Enro resistance could be compressed into a single mating experiment, in which Enro^R^ alleles were co-transferred from resistant to susceptible mycoplasma cells. Such event required the physical contact and a form of sexual competence of the pair [5,9], as well as one partner being already highly resistant. Independent mating experiments generated progenies with chimeric genomes made of the 5632-recipient chromosome in which sequences of the resistant PG2^E10^-donor were transferred and recombined at homologous loci. Under the antimicrobial selective pressure, all transconjugants displayed the mutated *parE-parC* operon or/and the mutated *gyrA* of the donor, but resistance *per se* (from 4 up to 250-fold-increase in resistance) was reached only when both mutated loci were co-transferred. These data are in agreement with conclusions drawn from the analyses of co-evolved lineages (see above): alteration of both the topoisomerase IV and the DNA gyrase subunit A is critical for mycoplasmas’ quinolones resistance.

Mutations not previously detected in neither of the parents even as a minor population, were observed in the transconjugants having acquired *parE-parC* donor sequences (corresponding to *parE*_390_, *parE*_423_ and *parE*_625_) (Fig 4A). The mutation affecting *parE*_390_ is also emerging in the donor strain after 40 serial passages in medium without Enro (Fig 4B), raising the question of whether *parE* mutations (i) were pre-existing in the parent population at undetectable levels and were preferentially selected after mating or (ii) occurred *de novo* during or just after mating. It is interesting to note that in the donor strain, while the *parE*_390_ mutation emerged over passages, the mutations *parE*_112_ and *parC*_84_ were conversely being replaced by wild type (wt) sequences. Whether *parE*_390_ is being beneficial to the transconjugants in the context of the experiment, either towards resistance or fitness, is not known.

Surprisingly, none of the selected transconjugants reached the MIC of the donor, most likely because none displayed the exact combination of *parE-parC* and *gyrA* mutations found in the predominant PG2^E10^ population. Whether such transconjugants did arise during mating but were outcompeted by others is one possible explanation. An interesting observation is that all transconjugants carried the mutated *gyrA* or the wt-*gyrB* of the donor, none having inherited both genes from a single parent. While the two strains, 5632 and PG2, encode very similar GyrA and GyrB products these are not strictly identical, with 99.2 and 98.6% identity respectively (S5 Table). Since the DNA gyrase is composed of two GyrA and two GyrB subunits, all transconjugants expressed a modified version when compared to that of the recipient cell prior to mating. Whether this provided an advantage in the context of our experiment, or whether it reflects an epistatic phenomenon [50,51] remains to be addressed.

The most unexpected outcome of this study was the extent of combinatorial variation obtained after mating. Mycoplasma Chromosomal Transfer (MCT) was initially shown to differ from classical Hfr- or *oriT*-mediated transfer in that it affects nearly every position of the genome with equal efficiency [9]. Because NGS data had been obtained using pools of transconjugants, MCT was then thought to be limited to the transfer of one or two proximate loci in between two cells. Here, analyses of individual transconjugants revealed a much complex picture with the simultaneous transfer of small and large fragments distributed around the genome (Fig 3A). Indeed, this phenomenon created within a single step a set of totally new genomes that were a combinatorial blend of the two parents. Thanks to the significant differences in genome sequence existing between the two parental strains (average 1 variation every 26 nt), transconjugant genomes could be reconstructed with a high level of precision, revealing that besides the gain and loss of entire genes, MCT also generated chimeric genes. Overall, MCT affected from 6 to 17% of the genome regardless of whether these encoded housekeeping or accessory gene functions.

An average of 18 donor-fragments co-exists in the new transconjugant genomes, of which only 2 carried the selectable Enro^R^ mutations. This implies (i) that a large amount of unrelated fragments silently co-transferred along with the selectable marker, some of which may confer the cell with new, yet unpredictable phenotypes and (ii) that a large proportion of mosaic genomes have not been selected and that most likely, the combinatorial possibilities of conjugative MCT are endless. Because MCT introduces variation instantly, one limitation of this phenomenon is the viability and adaptability rate of the resulting chimeric cells. Although both parents are of the same species, the overall success of the transconjugants depends on how well the donor and new chimeric genes interact with the remaining recipient’s genes in a particular environment [52]. For a few generations, the cell may have to cope with multimeric enzymes or products which sub-units are not of a perfect match depending of the protein turn-over of the recipient cell. To a lower extent, this situation resembles genome transplantation used to engineer mycoplasmas and thus faced a number of similar issues [53]. Although several transconjugants turned out to be highly resistant to Enro, their initial selection could only be achieved in low concentration of Enro (0.25 μg.mL^-1^). This raised the question of whether growth on selective media was impaired because of the low turnover of recipient wt GyrA/ParC or because of a synergistic effect of the Enro with the gentamicin used for selecting transconjugants.

Incorporating large amount of incoming donor DNA had a fitness cost for most transconjugants but not all. Surprisingly, this was counterbalanced after a few passages in media with even one transconjugant ending with a higher fitness than the recipient or the donor cell. In contrast, passaging had no effect on the fitness of Enro^R^ spontaneous mutants derived from the donor (Fig 5) suggesting that new genome configurations may require a certain period of time for fine-tuning. In search for compensatory mutations, comparative analyses of WGS before and after passages were conducted that indicated the emergence of subpopulations with an overall low number of mutations, none of which could explain the improved fitness. Quantifying pathogen fitness in its entire life cycle is not trivial [54] and whether transconjugants selected in this study perform better than their parents in the animal host remains to be addressed.

Clues on the impacts of MCT were provided by our earlier *in silico* work that revealed massive HGT in between phylogenetically distant ruminant *Mycoplasma* spp. [55]. Loci that were exchanged in *M*. *agalactiae* accounted for 18% of its genome and often encompassed gene cluster with highly conserved organisation that were distributed around the genome [55]. Rather than successive independent HGT events, this picture might reflect the concomitant transfers of multiple unrelated fragments during mating. Within the ruminant host, *M*. *agalactiae* and some members of the *M*. *mycoides* cluster are often re-isolated from a same organ where they co-habit [14], a prerequisite to conjugative transfers. Throughout the process of infection, these populations have to face a series of bottlenecks applied by the host-response and the host-hostile environment. The mycoplasma minimal cell may be particularly vulnerable to the deleterious effect of Muller’s ratchet due to its limited genetic content and lack in DNA repair components. MCT may provide these organisms with a means to rescue their injured genomes by restoring deleted or inactivated genes. Yet, the repertoire of mosaic genomes produced in the host is likely to be limited by a low MCT frequency, although some parameters such as stress may trigger the phenomenon, and the viability of the chimeric genomes within the hostile host-environment.

While sharing the same ecological niche is an obvious facilitator of HGT, MCT was shown in *M*. *agalactiae* to rely on ICE, most likely because these conjugative transfers being dependent on the ICE-encoded conjugal pore [3,5,9]. Although ICE occurrence varies among strains of a same species [25,56], conserved ICE-elements have been detected in about 50% of the mycoplasma species with sequenced genome [25] suggesting that MCT might not be restricted to ruminant mycoplasmas but may occur in species that colonize man and swine. Horizontal chromosomal transfers that do not conform to the canonical Hfr- (or *oriT*-based) model are increasingly being reported [7,8,57,58] and mosaic genomes were recently described in *Mycobacterium smegmatis* as the result of Distributive Conjugative Transfer (DCT) [52]. As for MCT, the exact molecular mechanism driving these events remains to be fully elucidated.

Overall, our study unravelled the astonishing capacity of MCT to generate unlimited genome diversity. While this process may contribute to counteract the erosion of the small mycoplasma genome [59], it can also rapidly promote the mycoplasma short-term adaptability to changing environment. Our findings reinforce the central role played by HGT in promoting evolutionary adaptation but also challenge our view on the boundaries of bacterial species and on our capacity in predicting the emergence of new phenotypes.

## Materials and methods

### Bacterial strains and culture conditions

The PG2 clone 55–5 [57] and 5632 clone C1 [41], further referred as PG2 and 5632 for simplicity, were previously derived from the PG2 and the 5632 strains of *Mycoplasma agalactiae* respectively. The 5632 gentamicin-resistant clone (5632^G3^), designated as 5632^G^-3 in previous publication [9], was obtained by stable insertion at nucleotide 919899 of the gentamicin-resistance gene, *aacA-aphD* [9]. All strains were propagated at 37°C in SP4 medium [60] supplemented with 5 mM pyruvic acid (Sigma-Aldrich) and 45 μg.mL^-1^ cefquinome (cobactan 4.5%, MSD Animal Health) and, when needed, with enrofloxacin (Sigma-Aldrich) and/or gentamicin (Sigma-Aldrich) at specified concentrations.

Based on CFU counts taken at different times of the exponential growth phase, the doubling time (or generation time, G) of *M*. *agalactiae* PG2 55–5 was calculated to be equal to 3.3 ± 0.14 hours per generation (ca. 7.2 generations per 24h). The number of generations needed to generate the mutants and the transconjugants was estimated using this value as reference multiplied by their time of growth in broth medium only (the number of generations needed for a single cell to form a colony was not taken into account).

### Selection of spontaneous enrofloxacin-resistant clones under selective antibiotic pressure

Mycoplasma PG2 55–5 cells from a 1 mL of mid-exponential culture were centrifuged for 15 min at 8000 g at room temperature and re-suspended in SP4 medium containing 0.5 μg.mL^-1^ enrofloxacin. After 48h, 10 μL cultures of 10^8^ to 10^9^ CFU.mL^-1^ were plated on SP4 agar plates with increasing enrofloxacin concentrations (0.5 to 2 μg.mL^-1^). Colonies were only obtained on plates containing 0.5 μg.mL^-1^ enrofloxacin. They were picked and propagated in SP4 broth medium with the same antimicrobial concentration. This represented the first step of selection used in this study and constituted the basis of the lineages. One to 3 additional rounds of selection were similarly performed with increasing concentration of enrofloxacin (ranging from 0.5 to 32 μg.mL^-1^), with colonies growing on the highest concentration being picked and subjected to the next round. A total of 108 clones were obtained, among which 22 clones corresponding to 6 lineages were analysed. In parallel, PG2 55–5 cultures (10^8^ to 10^9^ CFU.mL^-1^) were propagated by serial passaging (dilution 1/50 or 1/100), in broth medium containing increasing enrofloxacin concentration (0.25, 0.5, 1 and 10 μg.mL^-1^) to generate the resistant PG2^E10^ population.

### Characterisation of spontaneous enrofloxacin-resistant mutants

Mutations in Quinolone Resistance Determining Region (QRDR) sequences of the target genes were identified by direct sequencing using the BigDye Terminator chemistry [61,62] and by whole genome sequencing. Direct sequencing of genomic DNA was performed at the genomic platform of Get-Purpan (Toulouse, France) using primers listed in S1 Table and genomic DNA extracted with chloroform, as previously described [63]. Of note, amino-acid positions of type II topoisomerases were numbered according to the *Escherichia coli* K-12 strain nomenclature, GyrA (AAC75291.1), GyrB (AAT48201.1), ParC (AAC76055.1) and ParE (AAA69198.1).

### Selection of enrofloxacin-resistant transconjugants by mating

Mating experiments were performed as previously described [5]. Briefly, the donor strain (PG2^E10^) and the recipient strain (5632^G3^) were grown individually in SP4 medium, during 24 h. The two cultures were mixed at a 5632^G3^:PG2^E10^ cell ratio of 1:10 for one experiment (T5) and 10:1 for the second (T6) and then centrifuged for 5 min at 8000 g at room temperature. Cells were re-suspended in SP4 medium, incubated during 16 h at 37°C and an aliquot of 300μl was plated in SP4 agar containing gentamicin (50 μg.mL^-1^) and different enrofloxacin concentrations (from 0.25 to 8 μg.mL^-1^). After several days of incubation at 37°C, single colonies were picked from plates with the highest antibiotic concentration before being propagated in SP4 liquid medium with the same concentration of enrofloxacin. Of note, colonies were only observed on solid media containing 0.25 μg.mL^-1^ of enrofloxacin, with the exception of one transconjugant, T5-5, which grew at 0.5 μg.mL^-1^. Mating experiments using PG2 55–5 (Enro^S^) and 5632^G3^ were used as negative control, to test the absence of enrofloxacin spontaneous resistant clones. The frequency of transconjugants was determined as the number of transconjugants, divided by the number of PG2^E10^ donor parental cells.

### Characterisation of potential transconjugants by PCR

PG2- or 5632-specific PCR assays were used to determine the parental origin of 11 genomic loci across the transconjugant genomes (S1 Table). Of these, 8 were previously described [9] and 3 were specifically designed for this study that targeted *parC*, *gyrA* and *gyrB*. PCR assays were conducted using genomic DNA extracted with the chloroform method [63] and primers listed in S1 Table. The presence and position of the 5632^G3^-specific gentamicin resistance marker (Gm) was confirmed by a specific PCR using one primer inside the marker and the other in the flanking chromosomal sequence (S1 Table). All PCR amplifications were performed according to the recommendations of the Taq DNA polymerase suppliers (M0267S, New England Biolabs).

### Determination of the enrofloxacin Minimum Inhibitory Concentrations (MICs)

The enrofloxacin MICs were determined according to the recommendation of Hannan 2000 [64] using the agar dilution method as previously described [32]. Briefly, 1 μL of each clone diluted to 10^4^−10^5^ CFU.mL^-1^ was spotted on agar plates containing serial two-fold dilution of enrofloxacin (from 0.0625 to 64 μg.mL^-1^). MIC assays were performed in triplicates for each clone, and the median value was retained. The MIC was defined as the lowest concentration of enrofloxacin that prevented visible growth after 5 days at 37°C while, in parallel 30 to 300 CFU were observed on the antimicrobial-free control plate. Based on Hannan 2000 [64], we considered in this study isolates with MIC of ≤0.5 μg.mL^-1^ as susceptible (Enro^S^) while MIC ≤1 μg.mL^-1^ and ≥2 μg.mL^-1^ corresponded to intermediate and resistant isolates (Enro^R^). Here, isolates with MIC ≥16 μg.mL^-1^ were further referred as being highly resistant.

### Fitness competition assays

A pairwise competition assay was performed to estimate the relative fitness of evolved strains versus their ancestor (i. e. transconjugants versus 5632^G3^ or spontaneous mutants versus PG2 55–5). For each pair, the evolved and the ancestor clones were mixed in SP4 medium at a 1:1 cell ratio (10^4^ CFU.mL^-1^). Serial dilutions of the starting (0h, T0) and final (18h, T18) co-cultures were plated on SP4 plates containing none or 4 μg.mL^-1^ of enrofloxacin. After 5 days at 37°C, the number of CFU was determined for the ancestor and the evolved clones. Fitness of each clone relative to its ancestor was calculated according to the equation: w = Fitness _evolved/ancestor_ = ln(evolved at T18/evolved at T0)/ln(ancestor at T18/ancestor at T0) [65,66]. Concerning the fitness of Enro^S^ Genta^R^ transconjugants with MIC ≤0.5 μg.mL^-1^, selection onto enrofloxacin solid media was obviously not feasible. These were then performed using 5632 as the ancestor and the gentamicin as selective antimicrobial (50 μg.mL^-1^). Their fitness ratio was then corrected by multiplying by 1.17, a value equal to the fitness ratio of 5632 versus 5632^G3^. At least three replicates were performed for each assay and the mean value and the standard deviation (SD) were calculated. A value of 1 indicated a fitness of the evolved strain similar to the ancestor (5632^G3^ or PG2 55–5), a ratio lesser than 1 or greater than 1 indicated a fitness-cost or -benefit for the evolved strain, respectively.

### Illumina whole genome sequencing and bioinformatic analyses

Genomic DNA was extracted from mycoplasma cells using the phenol-chloroform method [67]. Whole genome sequencing was performed at the GATC Biotech facility (Konstanz, Germany) using Illumina technology HiSeq (paired-end, 2x150 bp). An average of 2x10^7^ reads by mutants or transconjugants was obtained, corresponding to an average of 3100X for coverage depth. One exception is the PG2^E10^ population that was sequenced by the Genome-Transcriptome facility of Bordeaux (France) using HiSeq (paired-end, 2x100 bp, 1.6x10^7^ reads, coverage 1700X). All bioinformatics analyses were performed using the galaxy platform hosted by Genotoul, Toulouse, France (bioinfo.genotoul.fr) and default parameters unless specified (see workflow S6 Table). The reads of each clone (fastq file) were mapped on the reference genome *M*. *agalactiae* PG2 (NC_009497.1) or 5632 (NC_013948.1), using Burrows-Wheeler Aligner (BWA, MEM algorithms, Galaxy version 0.8.0) [68]. The quality of the alignments was controlled with Qualimap 2.2.1 [69]. Calling variant analyses were performed using successively RealignerTargetCreator, IndelRealigner, PrintReads and HaplotypeCaller of GATK3 (Galaxy version 3.5.0) for SNPs and indels detection [70]. Variations with a quality lower than 10000 were excluded (Filter VCF file tool, Galaxy Version 1.0.0). Variations were considered as (i) fixed when present in ≥95% of the reads or (ii) non-fixed when present in <95% of the reads, as a result of coexisting sub-populations [71]. The percentage of each variation was calculated using the ratio AD/DP (AD: Allelic depths for the reference and alternative alleles; DP: Approximate read depth) provided by GATK. Alignments (bam file) and variations (vcf file) were visualized using the Integrative Genome Viewer (IGV 2.3.93) [72], Artemis 16.0.0 [73] and ACT 13.0.0 [74]. Reconstruction of the composite genome of transconjugants (PG2/5632) was possible because of the frequent polymorphisms existing between PG2 and 5632, on average 1 variation every 26 nt calculated using Nucmer [75] (S2 Fig). This was performed by PG2 specific reads detection as follows: transconjugants reads were aligned on the 5632 genome, reads with mismatch were recovered (select lines tool, Galaxy version 1.0.1) and these reads were then aligned on the PG2 genome. Only reads with no mismatch and regions with a coverage higher than 15 reads were conserved. These mapped reads, corresponding to PG2 transferred regions, were manually curated using Artemis (S2 Fig, S6 Table). This consisted in removing (i) false-positive fragments (also present in the negative control 5632G3), (ii) the *vpma* and *hsd* gene families which ones spontaneously undergo high-frequency, intraclonal recombination in propagating population [41] and (iii) fragments having no SNPs based on Bam files. Of note, the absence of contaminations between DNA libraries was ensured by (i) treating separate DNA batches, (ii) by matching PCR genotyping with sequence data (S1 Fig) and (iii) by independent Sanger sequencing of regions containing mutations detected by WGS in quinolone target genes.

## Supporting information

S1 Fig Mapping of the transferred loci in transconjugants as defined by PCR and by WGS. (PDF)

S2 Fig Compiled sequence reads and alignment of the transconjugants and the two parental strains, PG2^E10^ and 5632^G3^, to the PG2 55–5 reference genome. (PDF)

S1 Table Primers used for Sanger sequencing and PCR assays. (XLSX)

S2 Table List of mutations detected in Enro^R^ mutants. (XLSX)

S3 Table List of acquired and lost genes in transconjugants. (XLSX)

S4 Table List of mutations in Enro^R^ mutants and transconjugants at P0, P10 and P40. (XLSX)

S5 Table Variations in enrofloxacin target enzyme between PG2 and 5632 strains. (XLSX)

S6 Table Galaxy workflows for variant analyses and for reconstructing transconjugant genomes. (XLSX)

S7 Table European Nucleotide Archive (EMBL-EBI) accession numbers. (XLSX)

## References

[1] Wiedenbeck J, Cohan FM. Origins of bacterial diversity through horizontal genetic transfer and adaptation to new ecological niches. FEMS Microbiol Rev. 2011;35: 957–976. 10.1111/j.1574-6976.2011.00292.x 21711367

[2] Ochman H, Lawrence JG, Groisman EA. Lateral gene transfer and the nature of bacterial innovation. Nature. 2000;405: 299–304. 10.1038/35012500 10830951

[3] Baranowski E, Dordet-Frisoni E, Sagné E, Hygonenq M-C, Pretre G, Claverol S, et al The Integrative Conjugative Element (ICE) of *Mycoplasma agalactiae*: Key Elements Involved in Horizontal Dissemination and Influence of Coresident ICEs. mBio. 2018;9 10.1128/mBio.00873-18 29970462 PMC6030558

[4] Burrus V, Pavlovic G, Decaris B, Guédon G. Conjugative transposons: the tip of the iceberg. Mol Microbiol. 2002;46: 601–610. 12410819 10.1046/j.1365-2958.2002.03191.x

[5] Dordet Frisoni E, Marenda MS, Sagné E, Nouvel LX, Guérillot R, Glaser P, et al ICEA of *Mycoplasma agalactiae* : a new family of self-transmissible integrative elements that confers conjugative properties to the recipient strain: Mycoplasma ICE on the move. Mol Microbiol. 2013;89: 1226–1239. 10.1111/mmi.12341 23888872

[6] Wozniak RAF, Waldor MK. Integrative and conjugative elements: mosaic mobile genetic elements enabling dynamic lateral gene flow. Nat Rev Microbiol. 2010;8: 552–563. 10.1038/nrmicro2382 20601965

[7] Blesa A, Baquedano I, Quintáns NG, Mata CP, Castón JR, Berenguer J. The transjugation machinery of *Thermus thermophilus*: Identification of TdtA, an ATPase involved in DNA donation. PLoS Genet. 2017;13 10.1371/journal.pgen.1006669 28282376 PMC5365140

[8] Boritsch EC, Khanna V, Pawlik A, Honoré N, Navas VH, Ma L, et al Key experimental evidence of chromosomal DNA transfer among selected tuberculosis-causing mycobacteria. Proc Natl Acad Sci U S A. 2016;113: 9876–9881. 10.1073/pnas.1604921113 27528665 PMC5024641

[9] Dordet-Frisoni E, Sagné E, Baranowski E, Breton M, Nouvel LX, Blanchard A, et al Chromosomal Transfers in Mycoplasmas: When Minimal Genomes Go Mobile. mBio. 2014;5: e01958–14. 10.1128/mBio.01958-14 25425234 PMC4251992

[10] Gray TA, Krywy JA, Harold J, Palumbo MJ, Derbyshire KM. Distributive conjugal transfer in mycobacteria generates progeny with meiotic-like genome-wide mosaicism, allowing mapping of a mating identity locus. PLoS Biol. 2013;11: e1001602 10.1371/journal.pbio.1001602 23874149 PMC3706393

[11] Husain F, Tang K, Veeranagouda Y, Boente R, Patrick S, Blakely G, et al Novel large-scale chromosomal transfer in *Bacteroides fragilis* contributes to its pan-genome and rapid environmental adaptation. Microb Genomics. 2017;3 10.1099/mgen.0.000136 29208130 PMC5729914

[12] Lederberg J, Tatum EL. Gene recombination in *Escherichia coli*. Nature. 1946;158: 558. 10.1038/158558a0 21001945

[13] Hochhut B, Marrero J, Waldor MK. Mobilization of plasmids and chromosomal DNA mediated by the SXT element, a constin found in *Vibrio cholerae* O139. J Bacteriol. 2000;182: 2043–2047. 10715015 10.1128/jb.182.7.2043-2047.2000 PMC101929

[14] Citti C, Blanchard A. Mycoplasmas and their host: emerging and re-emerging minimal pathogens. Trends Microbiol. 2013;21: 196–203. 10.1016/j.tim.2013.01.003 23419218

[15] Razin S, Yogev D, Naot Y. Molecular Biology and Pathogenicity of Mycoplasmas. Microbiol Mol Biol Rev. 1998;62: 1094–1156. 9841667 10.1128/mmbr.62.4.1094-1156.1998 PMC98941

[16] Rosengarten R, Citti C, Much P, Spergser J, Droesse M, Hewicker-Trautwein M. The changing image of mycoplasmas: from innocent bystanders to emerging and reemerging pathogens in human and animal diseases. Contrib Microbiol. 2001;8: 166–185. 11764733 10.1159/000060409

[17] Citti C, Nouvel L-X, Baranowski E. Phase and antigenic variation in mycoplasmas. Future Microbiol. 2010;5: 1073–1085. 10.2217/fmb.10.71 20632806

[18] Gautier-Bouchardon AV. Antimicrobial Resistance in Mycoplasma spp. Microbiol Spectr. 2018;6 10.1128/microbiolspec.ARBA-0030-2018 30003864 PMC11633602

[19] Citti C, Browing GF, Rosengarten R. Phenotypic diversity and cell invasion in host subversion by pathogenic mycoplasmas Mycoplasmas Molecular Biology Pathogenicity and Strategies for Control. Horizon Bioscience. Wymondham: Blanchard Alain, Glenn Browing; 2005 pp. 439–483.

[20] Gaurivaud P, Baranowski E, Pau-Roblot C, Sagné E, Citti C, Tardy F. *Mycoplasma agalactiae* Secretion of β-(1→6)-Glucan, a Rare Polysaccharide in Prokaryotes, Is Governed by High-Frequency Phase Variation. Appl Environ Microbiol. 2016;82: 3370–3383. 10.1128/AEM.00274-16 27037120 PMC4959233

[21] Sirand-Pugnet P, Citti C, Barré A, Blanchard A. Evolution of mollicutes: down a bumpy road with twists and turns. Res Microbiol. 2007;158: 754–766. 10.1016/j.resmic.2007.09.007 18023150

[22] Vasconcelos ATR, Ferreira HB, Bizarro CV, Bonatto SL, Carvalho MO, Pinto PM, et al Swine and Poultry Pathogens: the Complete Genome Sequences of Two Strains of *Mycoplasma hyopneumoniae* and a Strain of *Mycoplasma synoviae*. J Bacteriol. 2005;187: 5568–5577. 10.1128/JB.187.16.5568-5577.2005 16077101 PMC1196056

[23] Teachman AM, French CT, Yu H, Simmons WL, Dybvig K. Gene Transfer in *Mycoplasma synoviae*. J Bacteriol. 2002;184: 947–951. 10.1128/jb.184.4.947-951.2002 11807054 PMC134802

[24] Torres-Puig S, Martínez-Torró C, Granero-Moya I, Querol E, Piñol J, Pich OQ. Activation of *σ20*-dependent recombination and horizontal gene transfer in *Mycoplasma genitalium*. DNA Res Int J Rapid Publ Rep Genes Genomes. 2018; 10.1093/dnares/dsy011 29659762 PMC6105099

[25] Citti C, Dordet-Frisoni E, Nouvel LX, Kuo CH, Baranowski E. Horizontal Gene Transfers in *Mycoplasmas* (Mollicutes). Curr Issues Mol Biol. 2018;29: 3–22. 10.21775/cimb.029.003 29648541

[26] Lo W-S, Gasparich GE, Kuo C-H. Found and Lost: The Fates of Horizontally Acquired Genes in Arthropod-Symbiotic *Spiroplasma*. Genome Biol Evol. 2015;7: 2458–2472. 10.1093/gbe/evv160 26254485 PMC4607517

[27] Pereyre S, Sirand-Pugnet P, Beven L, Charron A, Renaudin H, Barré A, et al Life on Arginine for *Mycoplasma hominis*: Clues from Its Minimal Genome and Comparison with Other Human Urogenital Mycoplasmas. PLoS Genet. 2009;5 10.1371/journal.pgen.1000677 19816563 PMC2751442

[28] Xiao L, Paralanov V, Glass JI, Duffy LB, Robertson JA, Cassell GH, et al Extensive horizontal gene transfer in ureaplasmas from humans questions the utility of serotyping for diagnostic purposes. J Clin Microbiol. 2011;49: 2818–2826. 10.1128/JCM.00637-11 21697330 PMC3147716

[29] Waites KB, Lysnyansky I, Bébéar C. Emerging antimicrobial resistance in mycoplasmas of humans and animals Mollicutes: Molecular biology and pathogenesis. Caister Academic Pr Norfolk: Citti Christine and Browing Glenn F.; 2014 pp. 289–322.

[30] von Wintersdorff CJH, Penders J, van Niekerk JM, Mills ND, Majumder S, van Alphen LB, et al Dissemination of Antimicrobial Resistance in Microbial Ecosystems through Horizontal Gene Transfer. Front Microbiol. 2016;7 10.3389/fmicb.2016.00173 26925045 PMC4759269

[31] Levy SB, Marshall B. Antibacterial resistance worldwide: causes, challenges and responses. Nat Med. 2004;10: S122–129. 10.1038/nm1145 15577930

[32] Khalil D, Becker CAM, Tardy F. Alterations in the Quinolone Resistance-Determining Regions and Fluoroquinolone Resistance in Clinical Isolates and Laboratory-Derived Mutants of *Mycoplasma bovis*: Not All Genotypes May Be Equal. Appl Environ Microbiol. 2016;82: 1060–1068. 10.1128/AEM.03280-15 26637606 PMC4751855

[33] Kenny GE, Young PA, Cartwright FD, Sjöström KE, Huang WM. Sparfloxacin Selects Gyrase Mutations in First-Step *Mycoplasma hominis* Mutants, whereas Ofloxacin Selects Topoisonmerase IV Mutations. Antimicrob Agents Chemother. 1999;43: 2493–2496. 10508030 10.1128/aac.43.10.2493 PMC89506

[34] Reinhardt AK, Kempf I, Kobisch M, Gautier-Bouchardon AV. Fluoroquinolone resistance in *Mycoplasma gallisepticum*: DNA gyrase as primary target of enrofloxacin and impact of mutations in topoisomerases on resistance level. J Antimicrob Chemother. 2002;50: 589–592. 12356806 10.1093/jac/dkf158

[35] Hooper DC, Jacoby GA. Mechanisms of drug resistance: quinolone resistance. Ann N Y Acad Sci. 2015;1354: 12–31. 10.1111/nyas.12830 26190223 PMC4626314

[36] Hopkins KL, Davies RH, Threlfall EJ. Mechanisms of quinolone resistance in *Escherichia coli* and *Salmonella*: recent developments. Int J Antimicrob Agents. 2005;25: 358–373. 10.1016/j.ijantimicag.2005.02.006 15848289

[37] Redgrave LS, Sutton SB, Webber MA, Piddock LJV. Fluoroquinolone resistance: mechanisms, impact on bacteria, and role in evolutionary success. Trends Microbiol. 2014;22: 438–445. 10.1016/j.tim.2014.04.007 24842194

[38] Correia S, Poeta P, Hébraud M, Capelo JL, Igrejas G. Mechanisms of quinolone action and resistance: where do we stand? J Med Microbiol. 2017;66: 551–559. 10.1099/jmm.0.000475 28504927

[39] Yoshida H, Bogaki M, Nakamura M, Nakamura S. Quinolone resistance-determining region in the DNA gyrase *gyrA* gene of *Escherichia coli*. Antimicrob Agents Chemother. 1990;34: 1271–1272. 2168148 10.1128/aac.34.6.1271 PMC171799

[40] Yoshida H, Bogaki M, Nakamura M, Yamanaka LM, Nakamura S. Quinolone resistance-determining region in the DNA gyrase *gyrB* gene of *Escherichia coli*. Antimicrob Agents Chemother. 1991;35: 1647–1650. 1656869 10.1128/aac.35.8.1647 PMC245234

[41] Nouvel LX, Sirand-Pugnet P, Marenda MS, Sagné E, Barbe V, Mangenot S, et al Comparative genomic and proteomic analyses of two *Mycoplasma agalactiae* strains: clues to the macro-and micro-events that are shaping mycoplasma diversity. BMC Genomics. 2010;11: 86 10.1186/1471-2164-11-86 20122262 PMC2824730

[42] Glew MD, Papazisi L, Poumarat F, Bergonier D, Rosengarten R, Citti C. Characterization of a multigene family undergoing high-frequency DNA rearrangements and coding for abundant variable surface proteins in *Mycoplasma agalactiae*. Infect Immun. 2000;68: 4539–4548. 10899853 10.1128/iai.68.8.4539-4548.2000 PMC98368

[43] Marenda M, Barbe V, Gourgues G, Mangenot S, Sagne E, Citti C. A New Integrative Conjugative Element Occurs in *Mycoplasma agalactiae* as Chromosomal and Free Circular Forms. J Bacteriol. 2006;188: 4137–4141. 10.1128/JB.00114-06 16707706 PMC1482908

[44] Frost LS, Leplae R, Summers AO, Toussaint A. Mobile genetic elements: the agents of open source evolution. Nat Rev Microbiol. 2005;3: 722–732. 10.1038/nrmicro1235 16138100

[45] Koonin EV. Horizontal gene transfer: essentiality and evolvability in prokaryotes, and roles in evolutionary transitions. F1000Research. 2016;5 10.12688/f1000research.8737.1 27508073 PMC4962295

[46] Gruson D, Pereyre S, Renaudin H, Charron A, Bebear C, Bebear CM. In Vitro Development of Resistance to Six and Four Fluoroquinolones in *Mycoplasma pneumoniae* and *Mycoplasma hominis*, Respectively. Antimicrob Agents Chemother. 2005;49: 1190–1193. 10.1128/AAC.49.3.1190-1193.2005 15728924 PMC549269

[47] Almahmoud I, Kay E, Schneider D, Maurin M. Mutational paths towards increased fluoroquinolone resistance in *Legionella pneumophila*. J Antimicrob Chemother. 2009;64: 284–293. 10.1093/jac/dkp173 19474069

[48] Sutera V, Levert M, Burmeister WP, Schneider D, Maurin M. Evolution toward high-level fluoroquinolone resistance in *Francisella* species. J Antimicrob Chemother. 2014;69: 101–110. 10.1093/jac/dkt321 23963236

[49] Zhang G, Wang C, Sui Z, Feng J. Insights into the evolutionary trajectories of fluoroquinolone resistance in *Streptococcus pneumoniae*. J Antimicrob Chemother. 2015;70: 2499–2506. 10.1093/jac/dkv134 26031465

[50] Vogwill T, Kojadinovic M, MacLean RC. Epistasis between antibiotic resistance mutations and genetic background shape the fitness effect of resistance across species of *Pseudomonas*. Proc Biol Sci. 2016;283 10.1098/rspb.2016.0151 27170722 PMC4874708

[51] Wong A. Epistasis and the Evolution of Antimicrobial Resistance. Front Microbiol. 2017;8: 246 10.3389/fmicb.2017.00246 28261193 PMC5313483

[52] Derbyshire KM, Gray TA. Distributive Conjugal Transfer: New Insights into Horizontal Gene Transfer and Genetic Exchange in Mycobacteria. Microbiol Spectr. 2014;2 10.1128/microbiolspec.MGM2-0022-2013 25505644 PMC4259119

[53] Labroussaa F, Lebaudy A, Baby V, Gourgues G, Matteau D, Vashee S, et al Impact of donor-recipient phylogenetic distance on bacterial genome transplantation. Nucleic Acids Res. 2016;44: 8501–8511. 10.1093/nar/gkw688 27488189 PMC5041484

[54] Diard M, Hardt W-D. Evolution of bacterial virulence. FEMS Microbiol Rev. 2017;41: 679–697. 10.1093/femsre/fux023 28531298

[55] Sirand-Pugnet P, Lartigue C, Marenda M, Jacob D, Barré A, Barbe V, et al Being Pathogenic, Plastic, and Sexual while Living with a Nearly Minimal Bacterial Genome. PLoS Genet. 2007;3: e75 10.1371/journal.pgen.0030075 17511520 PMC1868952

[56] Tardy F, Mick V, Dordet-Frisoni E, Marenda MS, Sirand-Pugnet P, Blanchard A, et al Integrative Conjugative Elements Are Widespread in Field Isolates of Mycoplasma Species Pathogenic for Ruminants. Elkins CA, editor. Appl Environ Microbiol. 2015;81: 1634–1643. 10.1128/AEM.03723-14 25527550 PMC4325163

[57] Sapriel G, Konjek J, Orgeur M, Bouri L, Frézal L, Roux A-L, et al Genome-wide mosaicism within *Mycobacterium abscessus*: evolutionary and epidemiological implications. BMC Genomics. 2016;17: 118 10.1186/s12864-016-2448-1 26884275 PMC4756508

[58] Lesic B, Zouine M, Ducos-Galand M, Huon C, Rosso M-L, Prévost M-C, et al A natural system of chromosome transfer in *Yersinia pseudotuberculosis*. PLoS Genet. 2012;8: e1002529 10.1371/journal.pgen.1002529 22412380 PMC3297565

[59] Naito M, Pawlowska TE. Defying Muller’s Ratchet: Ancient Heritable Endobacteria Escape Extinction through Retention of Recombination and Genome Plasticity. mBio. 2016;7: e02057–15. 10.1128/mBio.02057-15 27329757 PMC4916391

[60] Tully JG. Culture medium formulation for primary isolation and maintenance of mollicutes Molecular and diagnostic procedures in mycoplasmology: molecular characterization. Academic Press San Diego: Tully J. G.; 1995 pp. 33–39.

[61] Heiner CR, Hunkapiller KL, Chen S-M, Glass JI, Chen EY. Sequencing Multimegabase-Template DNA with BigDye Terminator Chemistry. Genome Res. 1998;8: 557–561. 9582199 10.1101/gr.8.5.557 PMC310720

[62] Hudson P, Gorton TS, Papazisi L, Cecchini K, Frasca S, Geary SJ. Identification of a virulence-associated determinant, dihydrolipoamide dehydrogenase (lpd), in *Mycoplasma gallisepticum* through *in vivo* screening of transposon mutants. Infect Immun. 2006;74: 931–939. 10.1128/IAI.74.2.931-939.2006 16428737 PMC1360363

[63] Chen WP, Kuo TT. A simple and rapid method for the preparation of gram-negative bacterial genomic DNA. Nucleic Acids Res. 1993;21: 2260 8502576 10.1093/nar/21.9.2260 PMC309503

[64] Hannan PC. Guidelines and recommendations for antimicrobial minimum inhibitory concentration (MIC) testing against veterinary mycoplasma species. Vet Res. 2000;31: 373–395. 10958240 10.1051/vetres:2000100

[65] Wiser MJ, Lenski RE. A Comparison of Methods to Measure Fitness in *Escherichia coli*. PLoS ONE. 2015;10 10.1371/journal.pone.0126210 25961572 PMC4427439

[66] Gagneux S, Long CD, Small PM, Van T, Schoolnik GK, Bohannan BJM. The competitive cost of antibiotic resistance in *Mycobacterium tuberculosis*. Science. 2006;312: 1944–1946. 10.1126/science.1124410 16809538

[67] Sambrook J, Fritsch E, Maniatis T. Molecular cloning: A laboratory manual: Vol. 2 2. ed S.l.: Cold Spring Harbor; 1989.

[68] Li H, Durbin R. Fast and accurate short read alignment with Burrows-Wheeler transform. Bioinforma Oxf Engl. 2009;25: 1754–1760. 10.1093/bioinformatics/btp324 19451168 PMC2705234

[69] Okonechnikov K, Conesa A, García-Alcalde F. Qualimap 2: advanced multi-sample quality control for high-throughput sequencing data. Bioinformatics. 2016;32: 292–294. 10.1093/bioinformatics/btv566 26428292 PMC4708105

[70] McKenna A, Hanna M, Banks E, Sivachenko A, Cibulskis K, Kernytsky A, et al The Genome Analysis Toolkit: A MapReduce framework for analyzing next-generation DNA sequencing data. Genome Res. 2010;20: 1297–1303. 10.1101/gr.107524.110 20644199 PMC2928508

[71] Sun G, Luo T, Yang C, Dong X, Li J, Zhu Y, et al Dynamic Population Changes in *Mycobacterium tuberculosis* During Acquisition and Fixation of Drug Resistance in Patients. J Infect Dis. 2012;206: 1724–1733. 10.1093/infdis/jis601 22984115 PMC3488197

[72] Thorvaldsdóttir H, Robinson JT, Mesirov JP. Integrative Genomics Viewer (IGV): high-performance genomics data visualization and exploration. Brief Bioinform. 2013;14: 178–192. 10.1093/bib/bbs017 22517427 PMC3603213

[73] Rutherford K, Parkhill J, Crook J, Horsnell T, Rice P, Rajandream MA, et al Artemis: sequence visualization and annotation. Bioinforma Oxf Engl. 2000;16: 944–945. 10.1093/bioinformatics/16.10.944 11120685

[74] Carver TJ, Rutherford KM, Berriman M, Rajandream M-A, Barrell BG, Parkhill J. ACT: the Artemis Comparison Tool. Bioinforma Oxf Engl. 2005;21: 3422–3423. 10.1093/bioinformatics/bti553 15976072

[75] Kurtz S, Phillippy A, Delcher AL, Smoot M, Shumway M, Antonescu C, et al Versatile and open software for comparing large genomes. Genome Biol. 2004;5: R12 10.1186/gb-2004-5-2-r12 14759262 PMC395750

