# Supplementary material for: Mycoplasmas under experimental antimicrobial selection: The unpredicted contribution of horizontal chromosomal transfer
Source: PLoS Genet. 2019 Jan 22;15(1):e1007910. doi: 10.1371/journal.pgen.1007910 (PMC6358093; doi:10.1371/journal.pgen.1007910)
Supplement: S1 Fig — (PDF) [file pgen.1007910.s001.pdf]

S1 Fig

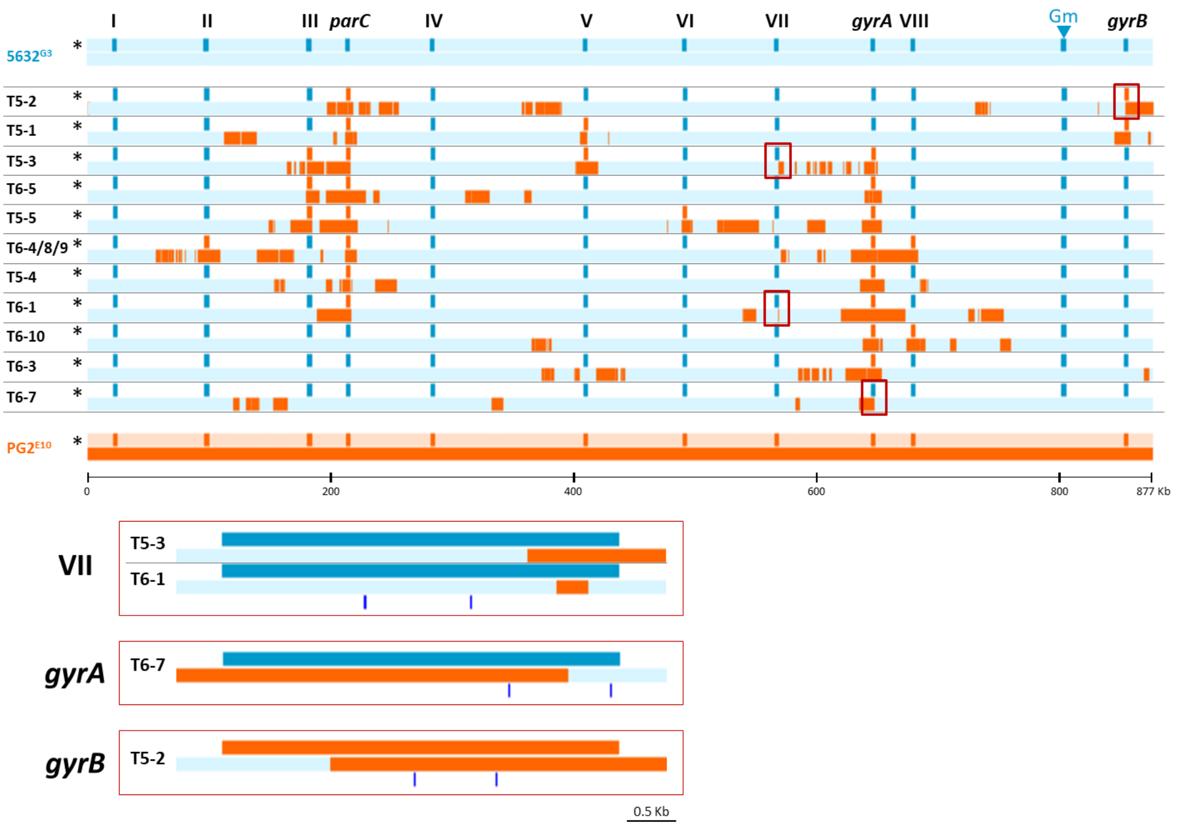

S1 Fig. Mapping of the transferred loci in transconjugants as defined by PCR and by WGS. Mosaic genomes reconstructed after WGS are represented by solid lines (starting with the OriC), with orange and blue lines respectively representing specific-PG2^{E10} or -5632^{G3} sequences. Results of the genotyping PCR assays (see Table S1 for primer list) are depicted above (*), with solid vertical bars representing positive, specific PCR products according to the colour code (orange being specific-PG2^{E10} and blue for 5632^{G3}). Loci targeted by specific-PG2 or -5632 PCR assays are indicated above, I to *gyrB*. Data boxed in red are enlarged below for better visualisation. Of note, PCR profiles of transconjugants T5-2 and T5-4 (mating experiment T5) shown above were also observed in transconjugants T6-2 and T6-1 (mating experiment T6) respectively, that were not fully sequenced and are not included above.
