# Supplementary material for: Mycoplasmas under experimental antimicrobial selection: The unpredicted contribution of horizontal chromosomal transfer
Source: PLoS Genet. 2019 Jan 22;15(1):e1007910. doi: 10.1371/journal.pgen.1007910 (PMC6358093; doi:10.1371/journal.pgen.1007910)
Supplement: S2 Fig — (PDF) [file pgen.1007910.s002.pdf]

S2 Fig

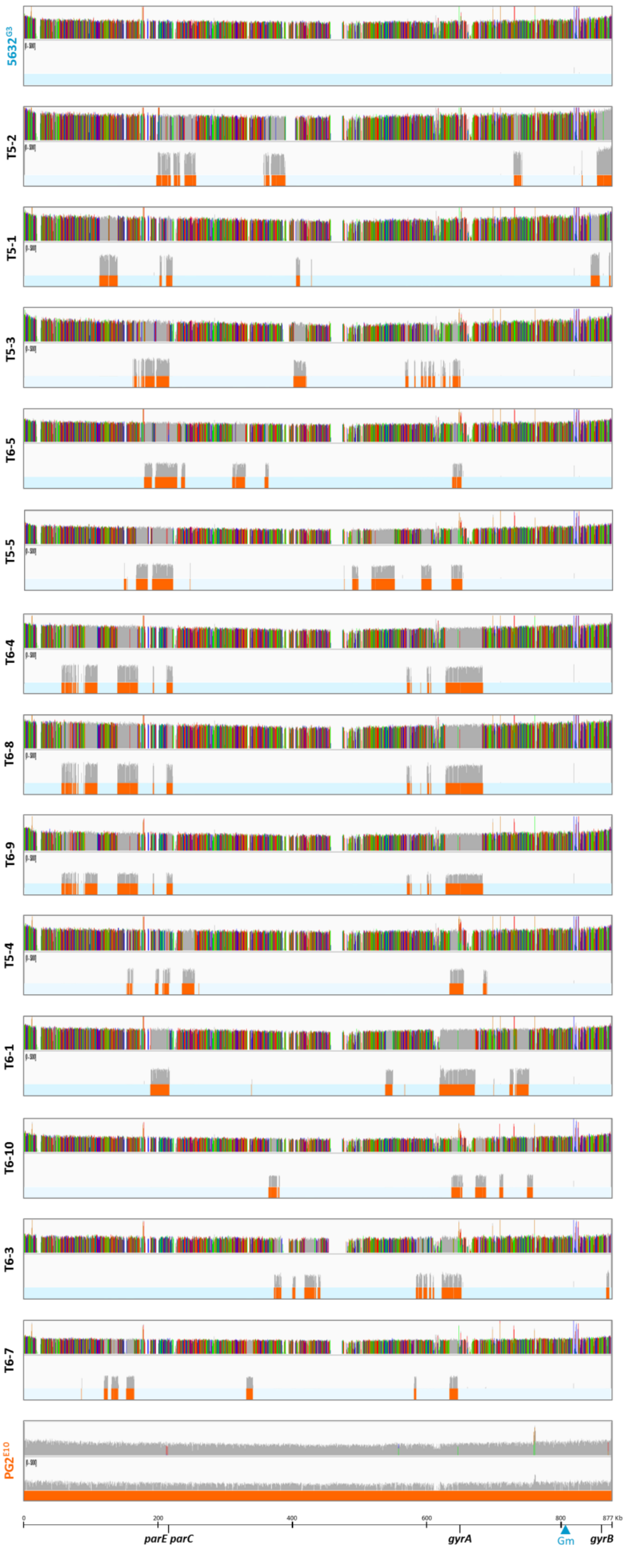

S2 Fig. Compiled sequence reads and alignment of the transconjugants and the two parental strains, PG2^{E10} and 5632^{G3}, to the PG2 55-5 reference genome.

For each strain or transconjugant, the upper line indicates the total reads and the lower line the PG2 specific reads extracted using the Galaxy workflow (S6 Table). These profiles are visualized with IGV at scale 5000. Sequences identical to PG2 55-5 are represented in grey and the presence of SNPs is indicated by colors, each color representing a different base. For transconjugants, colored SNPs indicate recipient sequences, while segments lacking SNPs define donor. A schematic of the genome (using Artemis) is provided for each transconjugant or strain as a filled bar (*) below the sequences, with the recipient segments in blue and donor in orange. The PG2^{E10} and 5632^{G3} markers and genes of interest relative to the PG2 55-5 strain are indicated at the bottom.
